# Identification of the RING-HCa E3 Ligase Gene Family and Functional Characterization of *StDRIP1* in Potato Drought Stress Response

**DOI:** 10.3390/cimb48080787

**Published:** 2026-08-02

**Authors:** Xiaoyuan Liu, Xingyu Zhou, Haoran Wen, Jin Gong, Ying Wang, Sa Song, Xiaodong Bai, Xiangyuan Shi, Yinyuan Wen, Meiqiang Yin

**Affiliations:** 1 College of Agronomy, Shanxi Agricultural University, Taiyuan 030031, China; lxy2385324147@163.com (X.L.); 13096272791@163.com (X.Z.); 13028010441@163.com (H.W.); 18536499974@163.com (J.G.); 18735941868@163.com (Y.W.); songsa0103@163.com (S.S.); 2 Key Laboratory of Potato Genetic Improvement and Germplasm Innovation in Shanxi Province, Shanxi Agricultural University, Taiyuan 030031, China; bxd5561@126.com (X.B.); sxy75@yeah.net (X.S.); 3 Institute of High Altitude Crop of Shanxi Agricultural University, Datong 037008, China

**Keywords:** potato, RING-type E3 ligases, HCa gene family, *StDRIP1*, drought stress

## Abstract

The RING-type E3 ubiquitin ligase plays a significant role in plant responses and adaptations to abiotic stresses such as drought. However, few studies have explored the role of E3 ubiquitin ligases in potato drought stress, especially *DRIP1*. In this study, 172 *StHCa* genes were identified across the potato genome. These genes were unevenly distributed on twelve chromosomes and divided into six subclades (group I–VI). The molecular weight of potato HCa proteins ranges from 5445.38 to 143,293.69 Da. More than half of them are acidic proteins and most are unstable. There are 161 hydrophilic proteins, and the subcellular localization analysis indicated that they were mainly located in the nucleus. The co-linearity analysis of *StHCa* genes showed that 172 genes underwent 40 tandem duplications and 28 segmental duplication events. Potato and tomato share a recent common ancestor and exhibit highly similar evolutionary trajectories. Promoter sequence analysis of the *StHCa* family identified abundant cis-acting elements associated with light signal transduction, hormone responses, plant growth and development, and abiotic stress responses. These results suggest that the *StHCa* genes may play important regulatory roles in different environmental signals and developmental stages. Expression profiling revealed that *StDRIP1* exhibited higher transcript levels in potato roots than in stems and leaves, and its expression was significantly induced by drought stress. Physiological phenotyping demonstrated that *StDRIP1*-overexpressing (OE) plants displayed reduced root growth compared with wild-type (WT) plants, with decreases in root length, total root area, total root volume, and root vitality. Under 20% PEG-6000-simulated drought stress, the root expression level of *StDRIP1* was higher in OE lines than in WT plants. Furthermore, *StDRIP1* overexpression suppressed the activities of antioxidant enzymes (POD, CAT, and SOD) and weakened their osmotic adjustment ability by reducing proline (Pro) accumulation. At the late stage of stress treatment (9 h), the SOD, POD, and CAT activities of OE plants were 5.0%, 19.9%, and 25.1% lower than those of WT plants, respectively. These physiological alterations exacerbated oxidative damage, as evidenced by increased malondialdehyde (MDA) content and elevated electrolyte leakage in OE plants. In summary, this study comprehensively characterized the *StHCa* gene family in potato, providing a valuable theoretical basis for elucidating the functional mechanism of *StDRIP1* in modulating drought stress responses. Collectively, *StDRIP1* acts as a negative regulator of potato drought tolerance through two primary mechanisms: (1) repressing root growth and weakening root vitality, thereby reducing the water absorption capacity of roots; and (2) diminishing antioxidant enzyme activities and impairing osmotic homeostasis, which further exacerbates oxidative damage under drought stress.

## 1. Introduction

Global food security and ecosystem stability face unprecedented challenges as drought becomes more frequent and severe [1]. Drought is the leading cause of agricultural production loss [2] by drastically hindering crop growth and development [3]. To survive under drought environmental conditions, crop plants have developed adaptive strategies that involve integrated molecular, cellular, and metabolic programs [4].

Ubiquitination is a widely existing post-translational protein modification in eukaryotes, whose core function is to degrade target proteins through the 26S proteasome pathway, thereby regulating key cellular processes such as signal transduction, growth and development, and stress responses [1,5]. Really Interesting New Gene (RING)-type E3 ligases have attracted much attention due to their large number and diverse functions. RING-HCa-type E3 ligases, whose fifth metal ligand is a Cys (C3HC4) with a shorter interval between the seventh and eighth metal-coordinating residues, are the largest class of RING-type E3 ligases and play important roles in plant growth and development, as well as responses to abiotic stress [6]. A total of 469 RING proteins (containing 474 RING domains) were identified in the tomato genome, among which 142 are of the RING-HCa type, accounting for the vast majority of the RING-HC type (142/163) [7]. Arabidopsis AtAIRP2, AtAIRP3, and AtAIRP4 (RING-HCa E3s) act as positive regulators of ABA-mediated drought stress responses [4,8]. For example, AtAIRP3/LOG2 is a positive regulator of the ABA-mediated drought and salt stress tolerance mechanism via the ubiquitination of RD21 [9]. OsDIS1 (Oryza sativa drought-induced SINA protein 1) is upregulated by drought and plays a negative role in drought stress tolerance through the transcriptional regulation of diverse stress-related genes and possibly through posttranslational regulation of OsNek6 (Ser/Thr protein kinase) in rice [10]. OsSADR1 contains RING-HC domains at the N- and C-termini with a nuclear localization signal and exhibits strong induction under salt, ABA, and drought exposure in rice roots [11]. ZmAIRP4 (Zea mays ABA-Insensitive RING Protein 4), was upregulated by ABA, polyethylene glycol, and sodium chloride, and ZmAIRP4-overexpressing Arabidopsis plants showed enhanced drought tolerance, which suggested that E3 ligase ZmAIRP4 is a positive regulator in the drought tolerance response pathway [12]. Although the functions of RING-HCa-type E3 ubiquitin ligases in plant stress responses have been well documented in numerous species, their biological roles in potato remain poorly understood.

DREB2A interacting protein (DRIP) acts as a RING-HCa E3 ubiquitin ligase to mediate DREB2A (Dehydration-Responsive Element Binding Protein 2A) protein ubiquitination degradation and thus negatively regulated drought stress responses by delaying drought-responsive gene expression [13]. Arabidopsis drought-inducible gene expression was significantly enhanced in the drip1 drip2 double mutant under dehydration stress [13]. Similar to the mechanism in Arabidopsis, VuDRIP functions as a ubiquitin ligase to negatively regulate VuDREB2A in cowpea (*Vigna unguiculata*), and it may also have other uncharacterized roles in stress and hormone signaling [14]. Wheat (*Triticum aestivum*) TaSAP5 (stress-associated protein) can interact with and ubiquitinate TaDRIP, as well as AtDRIP1 and AtDRIP2, leading to their subsequent degradation through the 26S proteasome pathway. Drought induces TaSAP5 expression in wheat, and TaSAP5 overexpression in Arabidopsis and wheat seedlings increased their drought tolerance [15]. These findings suggest that DRIP plays a critical role in crop drought responses.

Potato (*Solanum tuberosum* L.), a staple food crop with global significance, is widely cultivated for its high nutritional value and adaptability to diverse agronomic conditions [16,17]. However, its shallow root system and limited water transport capacity make it highly susceptible to drought stress, which has become a major constraint on potato production worldwide amid ongoing climate change [18,19]. Drought stress disrupts potato growth and development at multiple levels: it inhibits photosynthetic efficiency by inducing stomatal closure and reducing intercellular CO_2_ concentration [20], alters root architecture by suppressing lateral root formation under severe water deficit [21], and impairs tuber formation and quality accumulation [22]. In China, over half of potato-growing areas are located in arid or semi-arid regions, where insufficient precipitation leads to significant yield losses, threatening food security and farmers’ livelihoods [23]. Agricultural researchers have made tremendous efforts to identify key regulators in drought stress responses, providing new selections for constructing drought-tolerant potatoes [12]. However, the roles and molecular mechanisms of potato DRIP proteins in drought tolerance are still poorly understood.

In this study, we comprehensively analyzed the sequence composition, gene structures, cis-acting elements, chromosome positions, and gene replication events of 172 HCa family gene members in the potato. Evolutionary and homology analysis demonstrated that potato *StDRIP1* exhibits 54.93% sequence similarity with Arabidopsis *AtDRIP1/2*, suggesting a conserved function in modulating drought stress responses in potato. Tissue-specific expression profiling further demonstrated that *StDRIP1* is differentially expressed in potato roots, stems, and leaves. To dissect the drought-responsive function of *StDRIP1* in potato, we compared gene expression levels, root morphological traits, root vitality, antioxidant enzyme activities, and osmotic adjustment capacity between *StDRIP1*-overexpressing (OE) lines and wild-type (WT) plants under 20% PEG-6000-simulated drought stress. This study contributes to our understanding of the characteristics of the potato *StHCa* gene family and the physiological roles of *StDRIP1* in drought resistance.

## 2. Materials and Methods

### 2.1. Identification of the StHCa Gene Family and Analysis of the Physicochemical Properties

The complete genome sequence and annotation data of potato and *Arabidopsis thaliana* were retrieved from the PGSC version 6.01 (https://spuddb.uga.edu/dm_v6_1_download.shtml, accessed on 12 June 2023) and the TAIR (http://plants.ensembl.org/index.html, accessed on 12 June 2023). A combination of three distinct screening methods was employed to comprehensively identify all StHCa family members. Firstly, all Arabidopsis HCa proteins were used as queries for the BLASTP program (BLAST + V.2.13.0, E value < 1 × 10^−5^). Secondly, the Hidden Markov Model (HMM) profiles of the RING-HC domain (PF00097, PF13923, PF13920, and PF15227) were downloaded from the Pfam database (http://pfam.xfam.org, accessed on 25 June 2023), and HMMER 3.1 software (http://hmmer.org/download.html, accessed on 25 June 2023) was used to search and screen protein sequences that contain the RING-HC domain. Finally, the candidate members with RING-HC domains were manually verified through SMART (http://smart.embl-heidelberg.de, accessed on 28 June 2023) and the NCBI Conservative Domain Database (CDD). The physicochemical properties and subcellular localization of the *StHCa* genes were analyzed using Expasy-ProtParam (https://web.expasy.org/protparam/, accessed on 25 June 2023) and Cell-PLoc (http://www.csbio.sjtu.edu.cn/bioinf/plant-multi/, accessed on 29 June 2023), respectively.

### 2.2. Evolutionary Analysis of Ring-HCas

The ClustalW function in MEGA 12 software was utilized to align the HCa protein sequences of potato and *Arabidopsis thaliana*. The Neighbor-Joining (NJ) method with the P-distance model and pairwise deletion of gaps was employed to construct the evolutionary tree of these protein sequences. The confidence of each branch was evaluated through bootstrap (1000 replicates). The evolutionary tree was visualized using the online tool iTOL (https://itol.embl.de/, accessed on 25 June 2023).

### 2.3. Chromosomal Localization and Co-Linearity Analysis of the StHCa Family

The chromosome length data of potatoes and the physical location information of the *StHCa* gene were both derived from the potato genome annotation file. The chromosomal localization map of the members of the gene family was drawn using Tbtools II software, and the *StHCa* gene family members were sequentially named based on their positions on the chromosomes.

To understand the possible gene duplication events that occurred during the evolution of the *StHCa* gene family, a co-linearity map within the potato species was drawn using the Advanced Circos function in Tbtools II software. At the same time, the genome annotation files of five species, namely *Arabidopsis thaliana*, *Oryza sativa* (https://plants.ensembl.org/Oryza_sativa/Info/Index, accessed on 2 July 2023), *Solanum lycopersicum* (https://plants.ensembl.org/Solanum_lycopersicum_GCA_000188115.5cm/Info/Index, accessed on 25 June 2023), *Nicotiana tabacum* (https://plants.ensembl.org/Nicotiana_attenuata/Info/Index, accessed on 2 July 2023), and *Capsicum annuum* (https://plants.ensembl.org/Capsicum_annuum/Info/Index, accessed on 2 July 2023), were downloaded from the Ensembl database. The homology and co-linearity results between potatoes and different species were visualized using the Dual Systeny Plot function of TBtools.

### 2.4. Analysis of the Gene Structure, Conserved Motifs, and Promoter Cis-Acting Elements of the StHCa Family

To understand the structure of the *StHCa* gene, the domain structure of the *StHCa* gene protein sequence was systematically analyzed using the NCBI batch CD-search online tool (https://www.ncbi.nlm.nih.gov/Structure/bwrpsb/bwrpsb.cgi, accessed on 4 July 2023). At the same time, the conserved motifs of the *StHCa* gene protein sequence were analyzed through the MEME online website (https://meme-suite.org/meme/tools/meme, accessed on 4 July 2023), with the motif number set to 10 and other parameters set to default. The *StHCa* gene structure domain and conserved motifs were visualized using the gene structure view in Tbtools II. The software was also used to extract the promoter sequence upstream of the *StHCa* gene CDS of 2000 bp, and the trans-acting elements were analyzed and visualized.

### 2.5. Experimental Materials and Drought Stress

For generating the StDRIP1 transgenic potato, the full-length cDNA of StDRIP1 was inserted into the pBWA (V)KS vector and transformed into potato (Jinshu 16) stem segments using the Agrobacterium (GV3101)-mediated method. In vitro-grown potato seedlings of the cultivar Jinshu 16 and StDRIP1-transformed plants were cultured on MS medium and grown in the culture room (16-h-light/8-h-dark photoperiod at 25 ± 1 °C). Twenty-five-day-old seedlings in vitro were transferred to hydroponic culture with Hoagland solution for 20 days. Subsequently, the plants were transferred to Hoagland solution containing 20% PEG-6000 (*w*/*v*, ψ_s_ = 0.6 Mpa) to simulate drought stress and sampled at 0, 3, 6, and 9 h after treatment. The samples were rapidly frozen in liquid nitrogen and stored at −80 °C. Each treatment was set with 3 biological replicates.

### 2.6. qRT-PCR Analysis

Total RNA extraction was performed using the Total RNA Extraction Reagent (Coolaber, Beijing, China), according to the instructions. cDNA synthesis was carried out using the UnionScript First-Strand cDNA Synthesis Mix for qPCR (Genesand, Beijing, China). Specific primers for StDRIP1 and the reference gene Actin were designed using Primer Premier 6 software (PREMIER Biosoft International, Palo Alto, CA, USA), using cDNA as the template, and the qRT-PCR reaction was performed using the SYBR Green dye method (BBI, Shanghai, China) on the Bio-Rad CFX96 Real-time Fluorescence Quantitative PCR Instrument (Bio-Rad Laboratories, Hercules, CA, USA). The reaction system was 10 μL, containing 0.4 μL cDNA solution, 5 μL SYBR Premix EX Taq, 0.2 μL forward and reverse primers, and 4.2 μL ddH_2_O. The program conditions were as follows: pre-denaturation at 95 °C for 30 s; denaturation at 95 °C for 5 s; annealing at a temperature set according to the primer Tm value for 34 s; extension at 72 °C for 30 s; for a total of 39 cycles. The relative expression level of the gene was calculated using the 2^−ΔΔCT^ method, and the data were normalized. The primers used for qRT-PCR are listed in Table S2.

### 2.7. Measurement of Plant Physiological Indicators

The roots morphology of wild-type and transgenic plants was scanned and analyzed using a root scanner (Epson Perfection V850 Pro, Seiko Epson Corporation, Suwa, Nagano, Japan). The total root length, root total area, root total volume, and average diameter were measured. The root vitality was determined using the 2,3,5-triphenyltetrazolium chloride (TTC) reduction method, and the amount of TTC reduction represents the NADPH dehydrogenase activity and serves as an indicator of root activity. Therefore, the root vitality (µg·g^−1^·h^−1^) = TTC reduction amount/(root fresh weight × time). The leaf relative water content was measured by weighing. The root relative electrical conductivity was determined using an electrical conductivity meter. The chlorophyll content was measured by spectrophotometry. SOD activity was determined using the nitroblue tetrazolium method; POD activity was determined by the guaiacol method; CAT activity was determined using ultraviolet absorption; the MDA content was determined using the thiobarbituric acid method; the proline content was determined using the sulfosalicylic acid method; and the soluble protein content was determined using the Coomassie Brilliant Blue method. Each indicator was repeated 3 times, with at least 3 plants in each replicate.

### 2.8. Statistical Analysis

All experimental data were statistically analyzed using SAS.9.4 software. Analysis of variance (ANOVA) and Duncan’s New Multiple Range Test were used to determine significant differences. Graphs were plotted using Origin 2026 software. Error bars shown in the figures represent the standard deviation (SD).

## 3. Results

### 3.1. Identification and Physicochemical Characterization of the StHCa Gene Family

A total of 172 *StHCa* gene members were obtained and identified (Table S1). The physicochemical properties of these members, such as gene ID, amino acid sequence length, PI, molecular weight, hydrophobicity coefficient, instability index, and subcellular localization, were analyzed. All genes were named *StHCa1* to *StHCa172* according to their positions on the respective chromosomes. The amino acid lengths of StHCa proteins ranged from 50 aa to 1247 aa, among which only five proteins contained more than 1000 amino acids. The fatty acid coefficient ranged from 47.49 (StHCa38) to 108.13 (StHCa58). The molecular weight of StHCas ranged from 5445.38 Da (StHCa126) to 143,293.69 Da (StHCa52), with an average molecular weight of 45,273.51 Da. The isoelectric point (pI) ranged from 3.94 (StHCa167) to 9.74 (StHCa26). The instability index analysis revealed that there were 12 stable proteins, and the remaining proteins were unstable, accounting for 93%. The hydrophobicity analysis showed that 11 StHCas were hydrophobic proteins, and the rest were hydrophilic. Additionally, the subcellular localization analysis indicated that nine proteins were located in the cytoplasm, 22 proteins were in the chloroplast, three proteins were in the plasma membrane, StHCa53 was located in the cell wall, StHCa117 and StHCa132 were in the mitochondrion, and the remaining proteins were located in the cell nucleus. These results indicate the functional diversity of StHCa proteins.

### 3.2. Evolutionary Analysis of Potato HCa Gene Family Members

**Figure 1:**
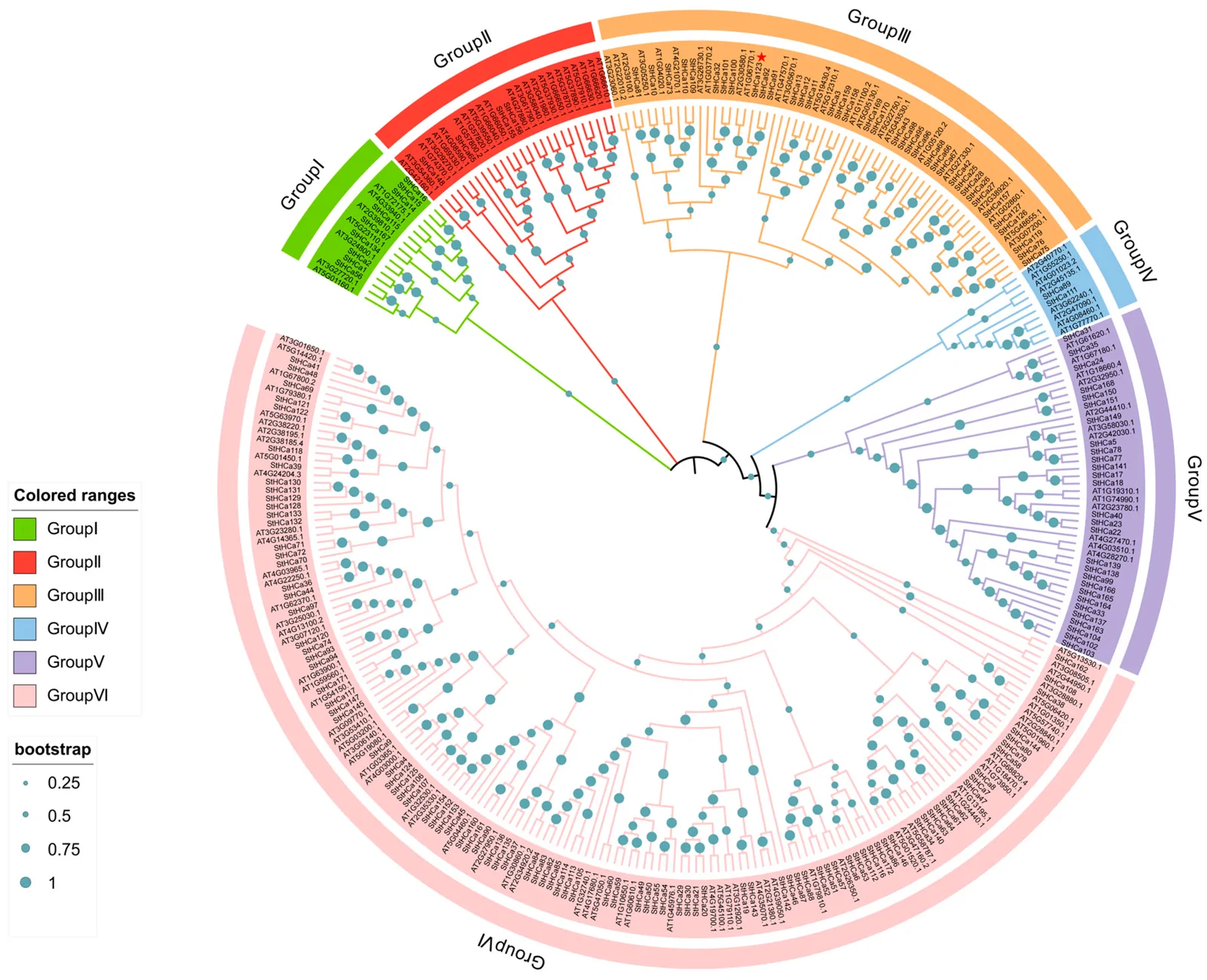
Evolutionary tree of potato and *Arabidopsis* RING-HCas. The red star represents the target gene (*StHCa123*).

In order to investigate the evolutionary relationship of *HCa* genes in potatoes, we constructed an evolutionary tree of HCa proteins from Arabidopsis (141 members) and potato (172 members). As shown in Figure 1, these proteins were divided into subclades, denoted as group I–VI. Group VI had the most StHCa proteins, up to 92, while groups III and V had 37 and 28 protein members, respectively, and the fourth group had the fewest members, only two genes. Potato PGSC0003DMT400038984 (StDRIP1) was 54.93% similar to AtDRIP1/2 (AT1G06770.1/AT2G30580.1) in group III, indicating that the protein may have similar functions as AtDRIP1/2, which negatively regulated drought stress by targeting DREB2A. PGSC0003DMT400057534 was similar to *AtCSU1* (AT1G61620.1) in group V. *AtCSU1* is an E3 ubiquitin ligase and negatively regulates COP1 protein accumulation and seedling photomorphogenesis. Potato PGSC0003DMT400039631 was similar to AT5G13530.1 (KEG) in group VI, indicating that the protein may have similar functions: responding to abscisic acid stimulus and stress defense.

### 3.3. Chromosomal Localization of the StHCa Gene Family

**Figure 2:**
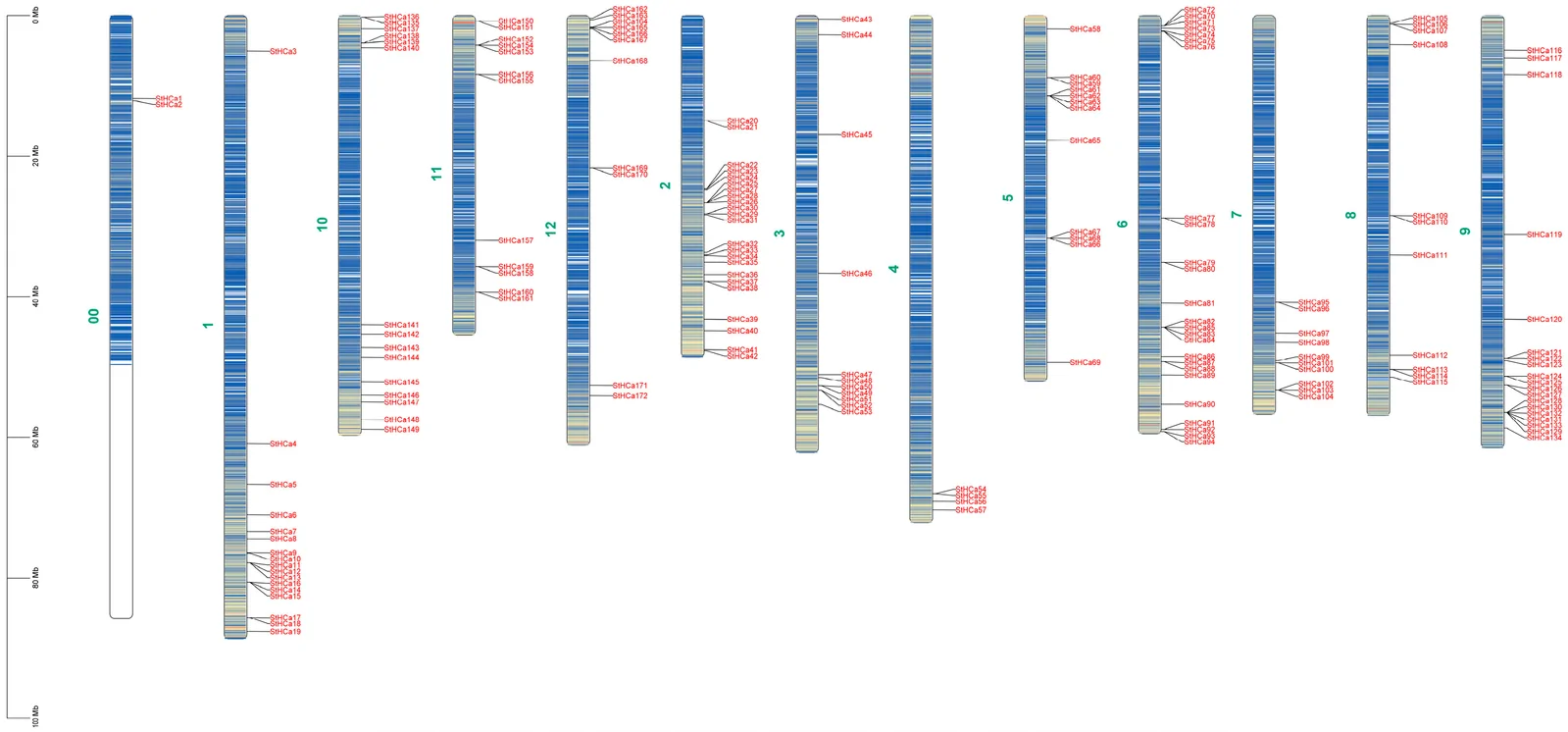
Location of *HCa* genes on potato chromosomes. Figure, the blue and red colors at the chromosome positions represent low gene density and high gene density respectively; the blank areas indicate that there is no gene distribution information in this genetic region.

Based on the physical mapping of the chromosomes of potato (*Solanum tuberosum* L.), we used Tbtools II software to display the 172 *StHCa* genes. One hundred and seventy *StHCa* genes are distributed across all 12 chromosomes, with each chromosome containing 4 to 25 genes (Figure 2). The number of *StHCa* genes on each chromosome varies significantly. Chromosome 6 contains the most family members (25 genes), while chromosome 4 has only four *StHCa* genes. Another two *StHCa* genes could not be anchored to any of the 12 pseudochromosomes during genome assembly (Chr00). Furthermore, *StHCa* genes underwent 40 tandem duplications across the chromosomes. Among these, chromosome 6 had the highest number of tandem duplication events, with up to eight gene clusters. Chromosomes 2 and 11 had five gene clusters, chromosomes 1, 5, 7, 8, and 9 each contained three gene clusters, chromosomes 3 and 10 each had two gene clusters, and chromosomes 4 and 12 each had one gene cluster. These results indicate that the distribution of *StHCa* genes across different chromosomes is not balanced, and this unbalanced distribution pattern may reflect the selective retention and functional differentiation of the *StHCa* family during evolution.

### 3.4. Analysis of the StHCa Gene Structure

To further characterize the potato *StHCa* family members, we constructed a phylogenetic tree based on protein sequences and complemented this with detailed analyses of the gene structure and conserved domains. The analysis of conserved domains revealed that the 172 *StHCa* family members contained 93 conserved domains, with most having the HC domain, which is consistent with the typical characteristics of the *StHCa* family (Figure S1B). Notably, HCa123 (StDRIP1) contains two conserved domains, RING-HC and Ubl1, with the RING-HC domain located at the N terminus, a pattern also observed in AtDRIP1/2. The analysis of gene structure results indicated that there were significant differences in the number of introns and exons among different family members of *StHCa*. The diversity of intron and exon structures usually plays a key role in the evolution of gene families and provides additional evidence to support the evolutionary groups. The number of exons in the *StHCa* family ranged from 1 to 18. There were 25 members of the *StHCa* gene that contained only one exon, and the gene with the most exons was *StHCa43,* with up to 18 exons. The number of introns and exons contained in different genes varied. Not all genes contained non-coding regions; for example, 20 *StHCa* genes lacked 5′UTR and 3′UTR (Figure S1A). Analyzing the gene structure, it was found that the structures of closely related genes were more similar, while the differences between genes with distant relationships were more obvious. It is speculated that different members of the *HCa* family may have independent or cooperative effects in the growth and development and defense processes of potatoes. This complex gene structure may provide more regulatory possibilities for post-transcriptional processing, further enriching the complexity and functional diversity of the proteome.

### 3.5. Conservation Analysis of the Conserved Motifs in the StHCa Gene Family

In terms of the conserved motifs, a total of 10 motifs were identified among the 172 *StHCa* genes. In terms of size, motif 1 was the shortest (8 aa), while motifs 3, 5, 6, 7, 8, and 9 were the longest, each having 50 aa. The motif average width was around 38 aa (Figure S3). The number of motifs in all *StHCa* genes ranged from 2 to 8 (Figure S2). Motifs 1 and 2 were present in all *StHCa* genes, indicating that motifs 1 and 2 have similar conserved positions and functions. The occurrence frequency of motif 4 was also relatively high, accounting for more than 80% of the total number of genes. The remaining different subfamily members added different types of motif structures on this basis. In contrast, motifs 5, 7, and 8 were selectively present in only a few *StHCa* proteins (Figure S2), suggesting that these motifs may have specific regulatory functions. An NCBI-CDD analysis showed that motif 4 harbors the RING-Ubox superfamily domain, whereas all other motifs possess the conserved characteristic ZF-C3HC4 domain (PF13920). This structural feature suggests that StHCa proteins participate in ubiquitination regulatory pathways and exert zinc-finger protein-mediated functions, such as transcriptional modulation, RNA binding, and protein–protein interactions.

### 3.6. StHCa Gene Correlation Analysis

**Figure 3:**
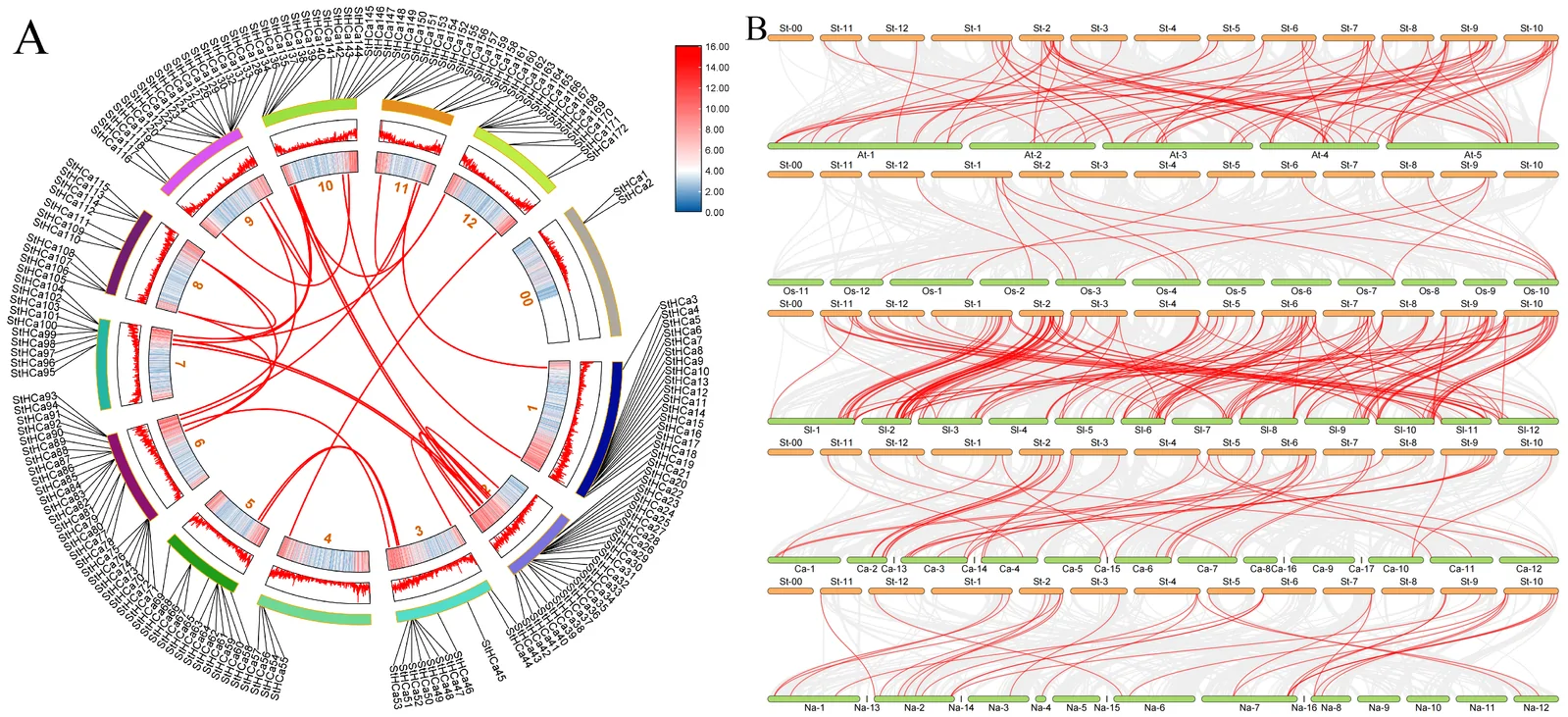
Intragenome collinearity of the *StHCa* gene family (**A**) and Collinearity analysis of *HCa* gene families between potato and *Arabidopsis thaliana*, *Oryza sativa*, *Solanum lycopersicum*, *Capsicum annuum*, *Nicotiana attenuata* (**B**). The gray line represents the co-linear blocks in the extensive genomic regions, while the red line indicates the direct homology relationship between the HCa gene and other species. In (**A**), different colors represent different chromosomes. The gradient color scheme uses blue to indicate low gene density and red to represent high gene density.

A species-specific and species-wide collinearity analysis was conducted on 172 members of the *StHCa* protein family. The species-wide collinearity results showed that 28 pairs of colinear genes were identified within the *StHCa* gene family. Among them, 23 pairs of colinear genes were distributed on different chromosomes, and five pairs of colinear genes were located on chromosomes 2, 9, and 11, respectively (Figure 3A). To further determine the homology relationship between the potato *HCa* gene family and other species, the potato *HCa* family genes were subjected to species-wide collinearity analysis, with five species including *Arabidopsis thaliana* L., *Oryza sativa*, *Solanum lycopersicum* L., *Capsicum annuum* L., and *Nicotiana attenuata*. The results revealed that there were 71 pairs of colinear gene pairs between potato and *Arabidopsis thaliana*, 14 pairs between potato and *Oryza sativa*, 148 pairs between potato and *Solanum lycopersicum* L., 38 pairs between potato and *Capsicum annuum* L., and 29 pairs between potato and *Nicotiana attenuata* (Figure 3B). The greatest number of collinear gene pairs was observed between potatoes and tomatoes, which belong to the same Solanaceae family and are genetically closely related to each other. This indicates that the collinearity of species is closely related to evolutionary changes. Notably, *StHCa123* (*StDRIP1*) shows collinear relationships with Arabidopsis AT2G30580.2 (DREB2A-interacting protein) and AT3G23060 (zinc ion-binding RING/U-box protein). Based on this collinearity evidence, we proposed hypotheses regarding the functional conservation of plant DRIP proteins, their potential ubiquitination substrate *StDREB2A*, and their regulatory roles within drought response pathways.

### 3.7. Analysis of the Cis-Acting Elements in the Promoter Region of the StHCa Gene

Cis-acting elements are essential for the transcriptional regulation of gene expression and modulate plant adaptability to diverse environmental conditions. To further explore the potential functions of the *StHCa* family genes, the 2000 bp upstream sequences of the CDS regions from 172 *StHCa* members were extracted using Tbtools II for the identification and analysis of promoter cis-acting elements (Figure S4). The results revealed that the cis-elements in *StHCa* promoters could be classified into three major categories: stress response, growth and development, and hormone regulation. The growth and development-related elements were associated with zein metabolism, flavonoid biosynthesis, meristem development, palisade mesophyll cell differentiation, and endosperm development. Hormone-responsive cis-elements mainly corresponded to abscisic acid, gibberellin, auxin, methyl jasmonate, and salicylic acid. Additionally, abundant cis-elements related to abiotic stresses, such as drought and low temperature, were also identified. Overall, light-responsive, drought-responsive, and gibberellin-responsive elements were highly enriched across *StHCa* family members. In contrast, only a few *StHCa* genes contained elements involved in endosperm-specific expression, meristem expression, and circadian rhythm regulation. Collectively, these findings suggest that *StHCa* genes are governed by complex regulatory networks and primarily participate in plant responses to abiotic stress.

### 3.8. StDRIP1 Transgenic Potato Plants Generation and StDRIP1 Expression Analysis

To further investigate the response of *StDRIP1* (homologous to *AtDRIP1/2*) to drought stress, transgenic potato plants were generated and screened. Following a 3-week cultivation period on MS medium, tissue samples were collected from the transgenic potato lines for genomic DNA extraction. Based on the kanamycin resistance marker, specific primers were designed to perform PCR-based positive identification of the transgenic plants. Clear target bands of the expected size were amplified in all tested transgenic lines, whereas no target fragment was detected in the wild-type potato plants (Figure S5).

**Figure 4:**
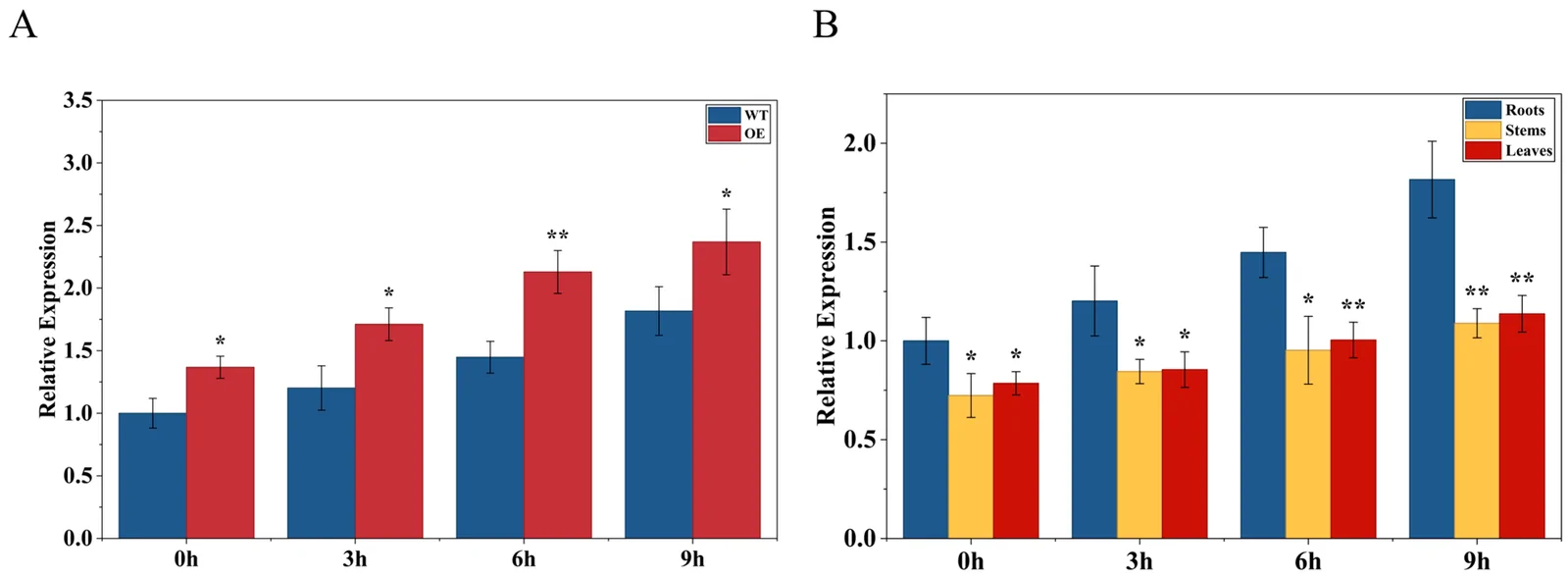
*StDRIP1* gene relative expression in the roots of WT and OE plants (**A**) and tissue-specific expression pattern in the wild-type potato plant (**B**) under drought stress. Twenty-day-old hydroponically cultivated seedlings exposed to drought stress with 20% (*w*/*v*) PEG-6000, and sampled at 0, 3, 6, and 9 h post-treatment. *, and ** indicate a significant difference at the 0.05, and 0.01 probability levels between WT and OE lines (ANOVA).

**Figure 5:**
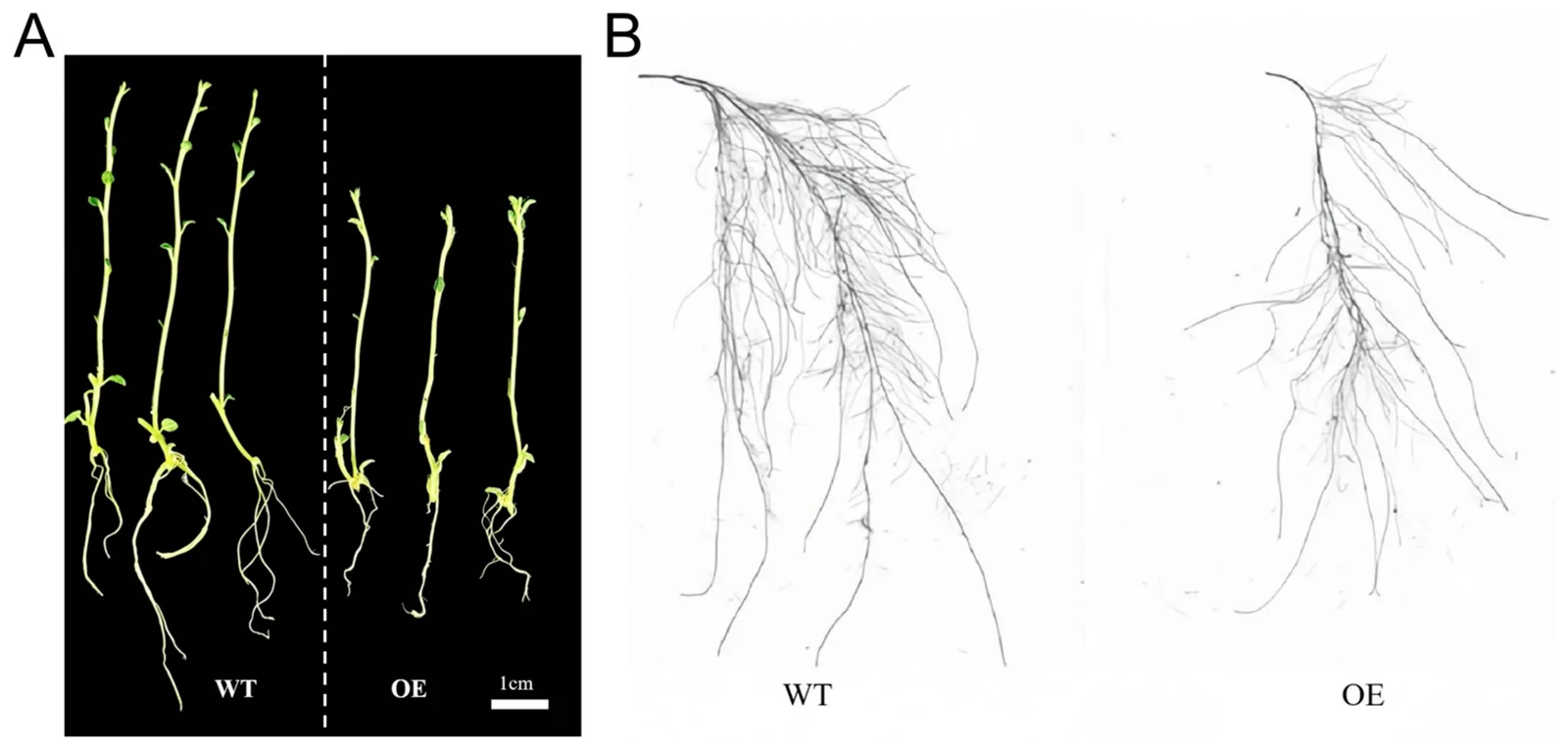
The morphology of potato StDRIP1-transgenic plants. Twenty-five-day-old seedlings in vitro (**A**) were transferred to hydroponic culture with Hoagland solution for 20 days, then the root morphology (**B**) of the WT and OE lines was scanned and analyzed using a root scanner.

*StDRIP1* transcript levels were significantly higher in overexpression lines than in wild-type plants, owing to extra exogenous *StDRIP1* copies continuously transcribed under the control of the 35S constitutive promoter. Additionally, drought stress further induced *StDRIP1* expression in overexpression lines in a time-dependent manner. The relative expression of *StDRIP1* peaked at 9 h post-stress treatment, with a value of 8.77, which was 1.7-fold greater than the baseline level at 0 h (Figure 4A). Overexpression of *StDRIP1* inhibited potato plant growth, resulting in a significant reduction in plant height. It had a more pronounced effect on the roots, manifesting as shorter root length, reduced total root length, total root area, total root volume, and average root diameter, and resulting in fewer lateral roots and a sparser root system in *StDRIP*-OE plants (Figure 5, Table 1).

Furthermore, the temporal and spatial expression patterns of *StDRIP1* in wild-type potato plants were examined by qRT-PCR. Transcript profiling revealed a distinct tissue-specific expression pattern of *StDRIP1*, with the highest abundance in roots, followed by stems and leaves. Under PEG-simulated drought stress, the expression of *StDRIP1* was significantly upregulated in all three tissues, peaking at 9 h post-treatment; the relative expression levels in roots, stems, and leaves at this time point were 1.8-fold, 1.5-fold, and 1.4-fold of those at 0 h, respectively (Figure 4B). These findings suggest that *StDRIP1* is a drought-inducible gene and may exerts a predominant regulatory role in the root system during drought stress responses in potato.

### 3.9. Changes in Root Vitality and Chlorophyll Content Under Drought Stress

**Figure 6:**
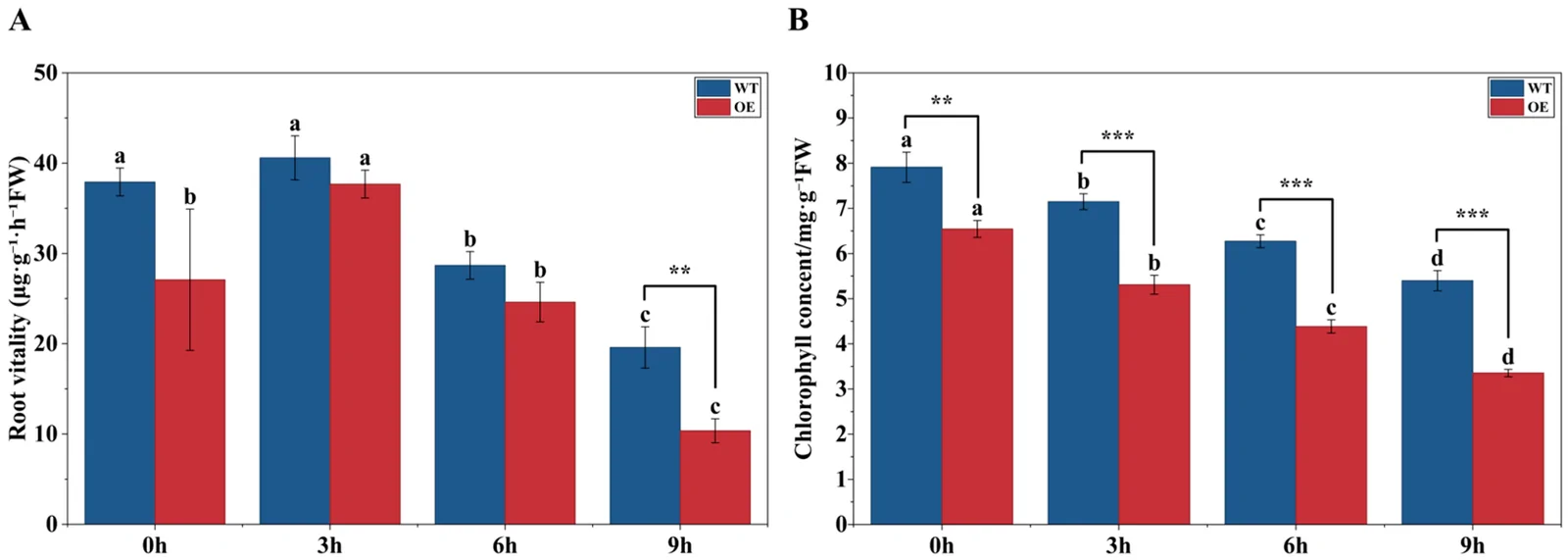
The changes in root vitality (**A**) and chlorophyll content (**B**) under drought stress. Different lowercase letters indicate significant differences among different stress durations (*p* < 0.05). **, and *** indicate a significant difference at the 0.01, and 0.001 probability levels between WT and OE lines (ANOVA).

Root vitality is an important indicator reflecting the overall metabolic strength of potato roots. Under normal conditions, the root vitality of the overexpressed plant line was 27.09 μg·g^−1^·h^−1^ FW, significantly lower than that of the WT (37.91 μg·g^−1^·h^−1^ FW). After 3 h of drought stress, root vitality increased, especially in the overexpressed plant line, where it increased significantly by 39.1% compared with that before the stress. This may represent a stress response that enhances water absorption capacity to maintain normal internal water balance under drought stress. As the treatment time extended, the damage caused by drought stress to root meristematic cells gradually increased, and root vitality sharply decreased; when the stress duration reached 9 h, root vitality in OE plants decreased to 10.36 μg·g^−1^·h^−1^ FW, which was significantly lower than that in WT plants (19.58 μg·g^−1^·h^−1^ FW) (Figure 6A). This indicates that StDRIP1 inhibits potato root NADPH reduction activity.

The chlorophyll content in *StDRIP1*-overexpressing (OE) lines was lower than that in wild-type (WT) plants under normal conditions (0 h). Upon exposure to drought stress, the chlorophyll content exhibited a gradual decline in both genotypes, with a more rapid reduction observed in the OE lines. At 9 h post-stress, the chlorophyll content in WT plants decreased by approximately 32%, whereas that in the OE lines decreased by 49% (Figure 6B). These results indicate that overexpression of StDRIP1 accelerates chlorophyll degradation or represses its de novo synthesis, thereby leading to severe impairment of photosynthetic capacity in potato plants under drought stress.

### 3.10. Analysis of Relative Water Content and Relative Conductivity of Leaves

**Figure 7:**
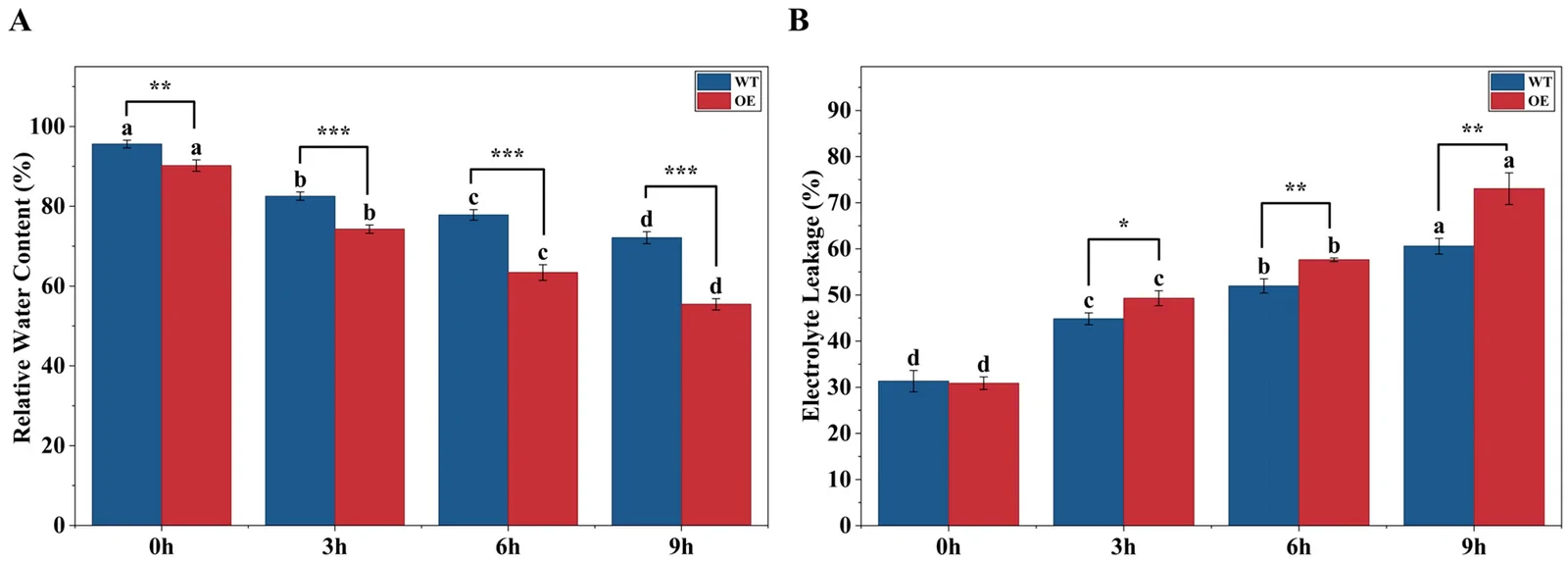
The variation of relative water content (**A**) and electrolyte leakage (**B**) under drought stress. Different lowercase letters indicate significant differences among different stress durations (*p* < 0.05). *, **, and *** indicate a significant difference at the 0.05, 0.01, and 0.001 probability levels between WT and OE lines (ANOVA).

Under drought stress, the relative water content (RWC) and relative conductivity (REC) of plant leaves showed a significant negative correlation and a synergistic change trend (Figure 7), jointly reflecting the degree of water deficit and the stability of cell membranes. With the extension of stress duration, the relative water content of both wild-type (WT) and overexpression lines (OE) of the plants continued to decrease, but the decline rate of the OE lines was significantly faster, and the decrease was greater. At 9 h of stress, the relative water content of the OE lines was only 55.42%, while that of the WT plants was 72.12%, indicating that overexpression of *StDRIP1* significantly reduced the plants’ water retention capacity and exacerbated water deficit in the plants. At the same time, the relative conductivity of both groups of plants increased with the increase in drought severity, and the increase in the OE lines was significantly greater than that in the WT plants. At 9 h of stress, the relative conductivity of the OE lines reached 73.04%, which was 20.6% higher than that of the WT lines, indicating that overexpression of *StDRIP1* exacerbated lipid peroxidation in the cell membrane, leading to membrane integrity damage and a large amount of electrolyte leakage.

In conclusion, overexpression of *StDRIP1* led to a decrease in the plants’ water retention capacity and aggravated cell membrane damage. The sharp decrease in leaf water content triggered cell dehydration, further damaging the cell membrane structure, whereas membrane damage exacerbated water loss, ultimately significantly reducing the plant’s drought resistance.

### 3.11. Analysis of Antioxidant Enzyme Activity and Malondialdehyde Content

**Figure 8:**
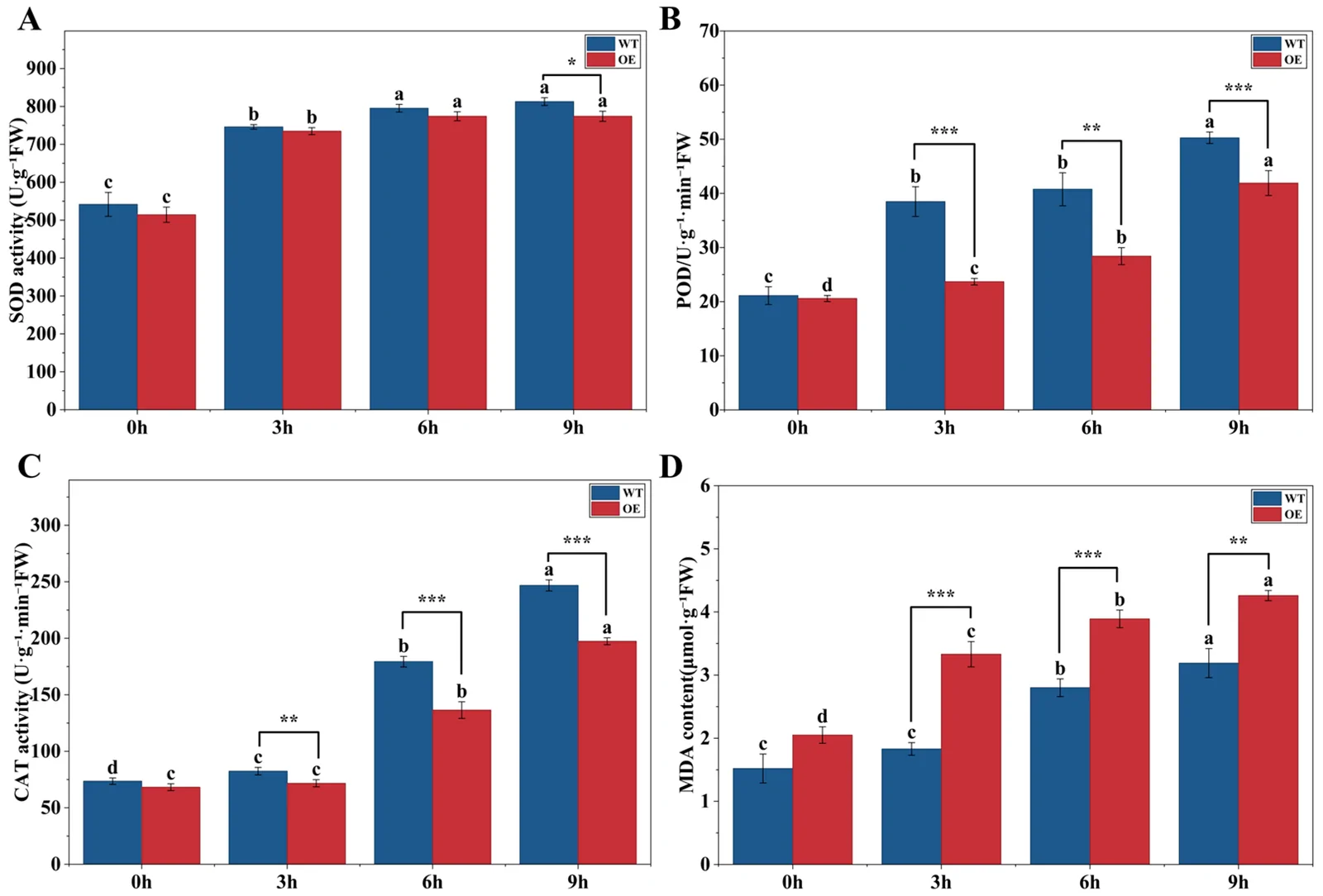
Antioxidant activity (**A**–**C**) and MDA content (**D**) under drought stress. Different lowercase letters indicate significant differences among different stress durations (*p* < 0.05). *, **, and *** indicate a significant difference at the 0.05, 0.01, and 0.001 probability levels between WT and OE lines (ANOVA).

Drought stress disrupts the internal environmental homeostasis of plants, thereby breaking their original metabolic balance. Harmful substances such as reactive oxygen species (ROS) and malondialdehyde (MDA) accumulate continuously in plants, damaging cell membrane structure and subsequently causing plant cell metabolic disorders. As the duration of drought stress increased, the activities of SOD, POD, and CAT in both wild-type (WT) and overexpression lines (OE) showed an upward trend, contributing to the elimination of ROS and alleviation of membrane lipid peroxidation. However, the increases in the activities of the three antioxidant enzymes in the OE lines were all lower than those in the WT, especially in the later stage of stress (9 h), when the SOD, POD, and CAT activities in the OE lines were 5.0%, 19.9%, and 25.1% lower than those of WT, respectively (Figure 8A–C). As a marker of plasma membrane peroxidation, the MDA content of WT and OE plants showed a continuous upward trend with increasing duration of drought stress. The MDA content of OE was significantly higher than that of WT at 9 h of stress, reaching 4.26 μmol·g^−1^ FW, which was 33.5% higher than that of WT (Figure 8D). This indicates that overexpression of StDRIP1 significantly inhibited the antioxidant enzyme activity of potato plants and exacerbated the accumulation of reactive oxygen species and membrane lipid peroxidation, thereby reducing the drought resistance of transgenic potatoes.

### 3.12. Analysis of Proline and Soluble Protein

**Figure 9:**
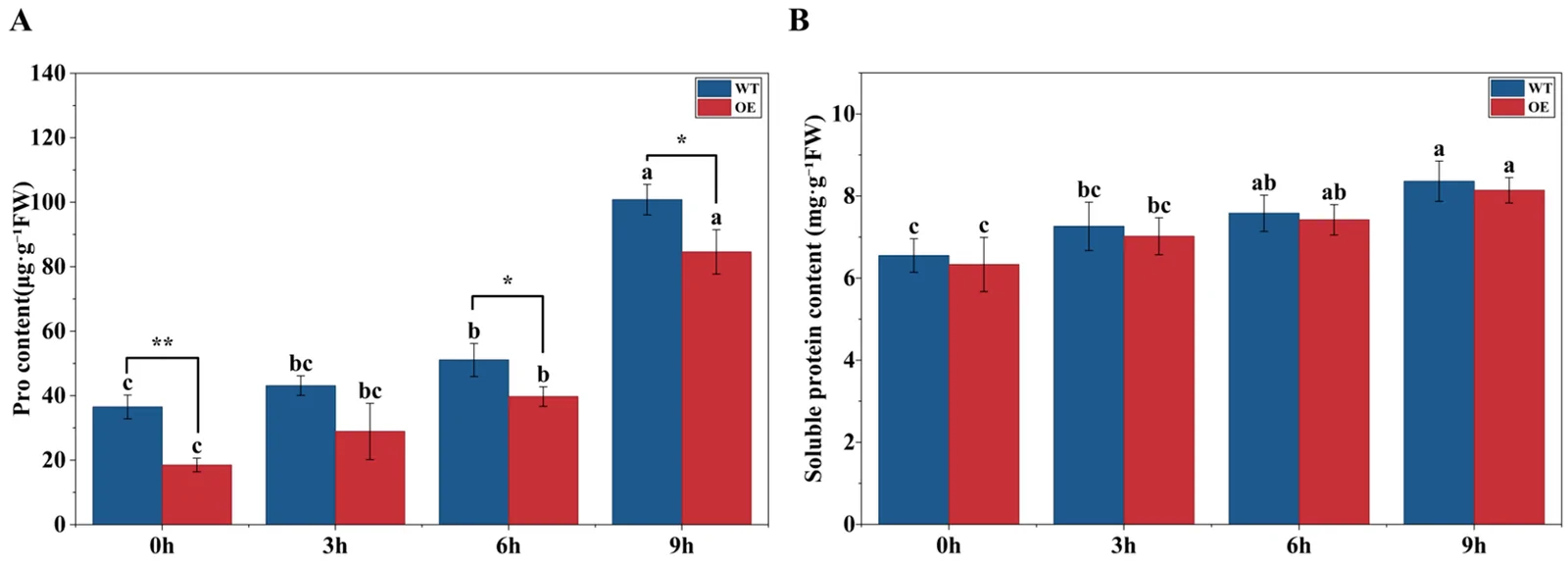
Proline (**A**) and soluble protein content (**B**) under drought stress. Different lowercase letters indicate significant differences among different stress durations (*p* < 0.05). *, and ** indicate a significant difference at the 0.05, and 0.01 probability levels between WT and OE lines (ANOVA).

Under drought stress, osmotic adjustment is an important physiological mechanism by which plants resist water deficiency. Proline (Pro) and soluble proteins, as core osmotic adjustment substances, maintain cellular osmotic pressure and stabilize cell membrane structure through their accumulation, and their content changes directly reflect the drought-adaptive ability of the plants. To clarify the regulatory role of *StDRIP1* in the potato osmotic adjustment system, this study measured the Pro and soluble protein contents of the wild-type (WT) and *StDRIP1* overexpression lines (OE) under different durations of drought stress (Figure 9). The results showed that the response of Pro to drought stress was relatively pronounced. Under normal growth conditions, the Pro content of the overexpression lines was lower than that of the wild-type plants, and as the duration of drought stress increased, the Pro content of both groups of plants showed a continuous upward trend. When the stress duration reached 9 h, Pro accumulation in both the overexpression and wild-type lines reached its peak. Although the Pro content of the OE line was still lower than that of the WT plants, its increase was significantly greater than that in the WT plants. This indicates that overexpression of *StDRIP1* inhibited the effective accumulation of Pro in the plants under normal conditions, weakening the ability of the plants to maintain cellular osmotic pressure and reduce water loss.

Soluble proteins are auxiliary osmotic adjustment substances that help plants to resist water deficiency and can maintain cellular osmotic pressure and stabilize the intracellular environment, alleviating the damage caused by drought stress. The results of this study showed that during the entire process of drought stress, the soluble protein contents of WT and OE lines showed a relatively small increasing trend, but there was no significant difference in soluble protein content between the two groups, indicating that the soluble proteins of the overexpression lines responded to potato drought stress, but their response was not strong.

In conclusion, the overexpression of *StDRIP1* inhibited the osmotic adjustment ability of potato plants by suppressing the effective accumulation of Pro and soluble proteins, causing cells to be unable to maintain normal osmotic pressure and resist water loss, further exacerbating the damage caused by drought stress in the plants.

## 4. Discussion

### 4.1. Identification and Analysis of StHCa Genes in Potato

In ubiquitin-mediated post-translational modifications, RING finger families have emerged as important E3 ligases involved in the regulation of biological processes, including embryogenesis, photomorphogenesis, circadian rhythm regulation, flowering, hormone signal transduction, and especially responses to biotic and abiotic stresses [24]. According to the amino acid type of the fifth metal-coordinating residue (ml5), RING domains are mainly divided into two major categories: RING-H2 type (ml5 is His) and RING-HC type (ml5 is Cys) [25]. Arabidopsis RING-HC E3 ligases were the second class with 186 domains in 477 RING-type E3 ligases. Within the RING-HC type, based on the length of the interval between the seventh and eighth metal-coordinating residues, it can be further classified into RING-HCa type (with a shorter interval) and RING-HCb type (with a longer interval) [6]. A total of 145 RING-HCa domains and 41 RING-HCb domains were identified in Arabidopsis [25]. Among 425 RING-type E3 genes in rice, 119 genes are categorized as RING-HC, with no distinction between HCa and HCb [26]. A total of 215 RING-HCa genes were identified in *Brassica rapa* [6], and 142 RING-HCa genes in tomato [7]. In the potato genome, a total of 172 *StHCa* genes were identified, which were unevenly distributed on twelve chromosomes. The molecular weight of potato *HCa* proteins ranges from 5445.38 to 143,293.69 Da. More than half of them are acidic proteins, and most are unstable proteins. There are 161 hydrophilic proteins, mainly located in the plasma membrane, cytoplasm and nucleus. Potato has various different gene sequences and physicochemical properties, indicating that the *StHCa* genes have different localizations and functions [6]. Chromosomal localization can reveal the distribution characteristics of genes in the genome. The co-linearity analysis of *StHCa* genes showed that 172 genes underwent 40 tandem duplications and 28 segmental duplication events. The duplication events promoted the diversity of the gene family. The co-linearity analysis with other species revealed a strong co-linearity relationship between potato and tomato, indicating a relatively close evolutionary distance and suggesting a recent common ancestor and similar evolutionary history between them.

Introns participate in the regulation of gene alternative splicing and essentially modulate gene functions. Structural analysis of potato *HCa* genes revealed distinct differences in the number and arrangement of exons and introns among family members. Nevertheless, genes clustered within the same subgroup share highly similar gene structures and conserved motifs, implying that this gene family has undergone relatively conserved evolutionary patterns. Cis-acting elements are specific DNA sequences that serve as binding sites for transcription factors. Their types and distribution are tightly associated with gene expression regulation, and thus have been widely adopted as key indicators for predicting putative gene functions [27]. Promoter sequence analysis of the *StHCa* gene family identified abundant cis-elements associated with light signaling, phytohormone responses, plant growth and development, as well as abiotic stress responses. These findings suggest that *StHCa* genes exert crucial regulatory functions in response to environmental stress signals and at multiple developmental stages. Taken together with previous studies, our results further demonstrate that *StHCa* genes are implicated in the transcriptional regulation of plant growth and stress adaptation [28,29,30].

### 4.2. StDRIP1 Overexpression Regulated Root Growth and Vitality in Potato

The plant root system is an essential part of the plant that functions as a sensor for abiotic and biotic signals, facilitates the uptake of nutrients and moisture from the soil, and acts as a support for the aerial parts of the plant. The developmental plasticity of the plant root system, which is affected by external signals and multiple internal hormonal cues, allows the root to adapt to the changing environment [31]. Regulated proteolysis by RING E3 ligases play an important role in root system development, either positively or negatively [32]. Arabidopsis transgenic plants that overexpress SINAT5 (RING finger-containing E3) show a reduction in the number of lateral roots, whereas plants that overexpress a dominant-negative mutant of SINAT5 produce more lateral roots [33]. The *XBAT32* gene is involved in lateral root development in Arabidopsis and *XBAT32* null mutant exhibits the delayed development of several above-ground organs because of the poor root system produced in the mutant plants [31,34]. Rice *OsDIS1* (C3HC4 RING-type E3 ligase) inactivation shifted resource allocation toward roots, increasing the root-to-shoot ratio, root length and surface area, thereby enhancing water foraging and drought plasticity [32]. Rice soil-surface rooting 1 (*SOR1*), which is a RING finger E3 ubiquitin ligase identified through the analysis of a rice ethylene-insensitive mutant *mhz2*/*sor1-2*, controls root-specific ethylene responses by modulating Aux/IAA protein stability. The *sor1-2* seedlings showed root-specific defects in ethylene-inhibited root growth and gravity response [35]. Rice Ring-HC E3 ligase gene *OsSIRHC-2*-overexpressing plants exhibited shorter plant lengths, lower shoot weights, higher root weights and higher chlorophyll content than WT plants [36]. Arabidopsis *drip1* and *drip2* mutants did not exhibit an apparent morphological phenotype, while plant development and growth of the *drip1 drip2* double mutant were significantly delayed and prolonged [13]. In this study, the expression level of the *StDRIP1* gene in the potato roots was higher than that in the stems and leaves, and the expression level of the *StDRIP1*-overexpressing plants was significantly higher than that of the non-transgenic potato roots. Root length, total root area, and total root volume of the *StDRIP1*-overexpressing plants were all lower than those in the WT plants (Figure 5, Table 1).

Root vitality, as a key parameter reflecting the growth status of plants, directly regulates the efficiency of water and mineral element absorption, and thereby affects the overall growth and development of the plants [37]. Root vitality is significantly positively correlated with the capacity to absorb water and nutrients, which is conducive to maintaining the normal physiological functions of plants [38]. Under drought stress conditions, the increase in root vitality of drought-resistant crop varieties is more pronounced [39]. The root vitality of the *StDRIP1*-overexpressing potatoes was significantly lower than that of the WT, and gradually decreased during drought stress (Figure 6), thereby reducing their ability to absorb water and nutrients. This ultimately led to reduced water content and inhibition of plant growth (Figure 7). These results suggest that *StDRIP1* reduces potato drought tolerance by inhibiting root growth and vitality [13,32].

### 4.3. StDRIP1 Reduced Drought Tolerance in Potato by Affecting Antioxidant Systems and Osmotic Regulation

Under adverse stress conditions, plants usually adjust their physiological metabolism actively to adapt to environmental pressures. Among these, activating antioxidant systems and accumulating osmotic adjustment substances to maintain redox homeostasis, alleviate oxidative damage, and preserve water balance, thereby ensuring normal physiological functions under drought stress [40]. Drought-induced Ring-HC E3 ligase genes have been identified, which effect physiological and molecular changes via ubiquitination. For example, *AtDRIP1* and *AtDRIP2* act as negative regulators in drought-responsive gene expression by targeting DREB2A to 26S proteasome proteolysis in Arabidopsis. Disruption of both *DRIP1* and *DRIP2* in double mutant (*drip1 drip2*) significantly increased the expression of a large number of dehydration stress genes, especially genes regulated by DREB2A [13]. Similarly, *OsDIS1* acts negatively on drought stress tolerance by post-transitional regulation of *OsNek6* (Ser/Thr protein kinase) in rice [10]. *Osdis1* mutant exhibited enhanced drought tolerance and better post-stress recovery, supported by improved chloroplast integrity and robust antioxidant defenses further mitigate oxidative damage, sustaining cellular homoeostasis [32]. *AIRP4* plays an active role in ABA-mediated drought stress response by regulating the drought-related downstream genes in maize and Arabidopsis [8]. In addition, Arabidopsis *AtAIRP2* and *AtAIRP3* act as a positive regulator of ABA-mediated drought stress responses [4]. After overexpression of *TaSADR1*, under drought treatment, the MDA content in transgenic Arabidopsis plants was higher than that in the WT plants, while the activities of POD, CAT and SOD, and proline content in the transgenic plants were lower than those in the WT plants [41]. Both AtSP1 and *AtSPL1* are C3HC4-type E3 ubiquitin ligases. *AtSP1* can promote the degradation of PEX13-PEX14, thereby reducing the plant’s antioxidant capacity. On the other hand, *AtSPL1* can prevent *AtSP1* from degrading PEX13-PEX14, thereby enhancing the activity of antioxidant enzymes in plants [42,43]. Greater accumulation of soluble sugars and proline makes *OsSIRHC-2* transgenic plants more resistant to salinity stress than WT plants [36]. The *TaSADR1*-overexpression Arabidopsis plants had lower proline content and exhibited reduced drought tolerance [41]. Potato *StDRIP1* exhibits 54.93% sequence similarity with Arabidopsis *AtDRIP1* and has a conserved function in modulating drought stress responses. Under 20% PEG-6000-simulated drought stress, the expression level of *StDRIP1* was significantly higher in the roots of overexpression potato lines than in wild-type plants. Overexpression of *StDRIP1* reduced the activities of antioxidant enzymes (POD, CAT and SOD) (Figure 8), and impaired osmotic adjustment capacity by decreasing proline (Pro) accumulation (Figure 9). These changes further aggravated oxidative damage, as reflected by increased malondialdehyde (MDA) content and elevated electrolyte leakage (Figure 8). Collectively, potato *StDRIP1* negatively regulates drought tolerance via modulating antioxidant defense systems and osmotic adjustment processes [13,32].

## 5. Conclusions

This study identified 172 RING-type E3 ubiquitin ligase *StHCa* genes family in potato and divided them into six subclades. The genes were unevenly distributed across twelve chromosomes. *StHCa* sequence composition, gene structures, cis-acting elements and chromosomal positions were analyzed. The *StDRIP1* promoter region contains multiple stress-responsive cis-acting elements related to drought and abscisic acid (ABA). It was more highly expressed in roots and was induced by osmotic stress. *StDRIP1* negatively regulates potato drought tolerance via (1) reducing water absorb ability by inhibiting potato root growth and vitality, (2) aggravating oxidative damage by reducing the activities of antioxidant enzymes and impairing osmotic adjustment.

Although these findings help clarify the physiological response of StDRIP1 to drought stress, a more detailed investigation would shed more light on the functional domains of StDRIP and their interacting partners in StDREB2A, including the generation of *StDRIP1* knockout mutants and the screening of downstream drought signaling pathways. In vitro ubiquitination assays are also needed to confirm whether StDRIP directly ubiquitinates StDREB2A for subsequent proteasome-mediated degradation. Further transgenic studies, for instance knocking down such negative regulators while overexpressing StDREB2A, would improve drought tolerance and provide deeper insights into the drought stress signaling mechanism in potato.

## Figures and Tables

**Table 1:** Comparison of root characteristics between WT and OE strains.

Roots Morphology	WT	OE
Total root length (cm)	966.19 ± 108.49	462.98 ± 78.66 **
Total root surface area (cm^2^)	70.02 ± 9.06	34.15 ± 11.29 *
Total root volume (cm^3^)	0.40 ± 0.06	0.18 ± 0.07 *
Average root diameter (mm)	0.23 ± 0.01	0.21 ± 0.01

Note: * and ** indicate a significant difference at the 0.05, and 0.01 probability levels between WT and OE lines (ANOVA).

## Data Availability

The original contributions presented in this study are included in the article and Supplementary Materials. Further inquiries can be directed to the corresponding authors.

## Supplementary Materials

The following supporting information can be downloaded at: https://www.mdpi.com/article/10.3390/cimb48080787/s1.
